# HDSNE a new unsupervised multiple image database fusion learning algorithm with flexible and crispy production of one database: a proof case study of lung infection diagnose In chest X-ray images

**DOI:** 10.1186/s12880-023-01078-3

**Published:** 2023-09-18

**Authors:** Muhammad Atta Othman Ahmed, Ibrahim A. Abbas, Yasser AbdelSatar

**Affiliations:** 1 Department of Computer Science, Faculty of Computers and Information, Luxor University, Luxor, 85951 Egypt; 2 Mathematics Department, Faculty of Science, Sohag University, Sohag, 82511 Egypt

**Keywords:** COVID-19, X-ray, Coronavirus, MD5, t-SNE, Data aggregation, Transfer Learning, Inception V3, Model production

## Abstract

Continuous release of image databases with fully or partially identical inner categories dramatically deteriorates the production of autonomous Computer-Aided Diagnostics (CAD) systems for true comprehensive medical diagnostics. The first challenge is the frequent massive bulk release of medical image databases, which often suffer from two common drawbacks: image duplication and corruption. The many subsequent releases of the same data with the same classes or categories come with no clear evidence of success in the concatenation of those identical classes among image databases. This issue stands as a stumbling block in the path of hypothesis-based experiments for the production of a single learning model that can successfully classify all of them correctly. Removing redundant data, enhancing performance, and optimizing energy resources are among the most challenging aspects. In this article, we propose a global data aggregation scale model that incorporates six image databases selected from specific global resources. The proposed valid learner is based on training all the unique patterns within any given data release, thereby creating a unique dataset hypothetically. The Hash MD5 algorithm (MD5) generates a unique hash value for each image, making it suitable for duplication removal. The T-Distributed Stochastic Neighbor Embedding (t-SNE), with a tunable perplexity parameter, can represent data dimensions. Both the Hash MD5 and t-SNE algorithms are applied recursively, producing a balanced and uniform database containing equal samples per category: normal, pneumonia, and Coronavirus Disease of 2019 (COVID-19). We evaluated the performance of all proposed data and the new automated version using the Inception V3 pre-trained model with various evaluation metrics. The performance outcome of the proposed scale model showed more respectable results than traditional data aggregation, achieving a high accuracy of 98.48%, along with high precision, recall, and F1-score. The results have been proved through a statistical t-test, yielding *t*-values and *p*-values. It’s important to emphasize that all *t*-values are undeniably significant, and the *p*-values provide irrefutable evidence against the null hypothesis. Furthermore, it’s noteworthy that the Final dataset outperformed all other datasets across all metric values when diagnosing various lung infections with the same factors.

## Introduction

COVID-19 began to be reported in late 2019 in response to an unusual increase in infected patients in Wuhan, China. The COVID-19 epidemic has already infected over 96 million people and claimed the lives of at least 2 million individuals worldwide [1], with very few parallels in history. The virus quickly spread around the world, initially through individual transmissions and then through community transmissions, becoming a major public health concern. Coronavirus strains possess a positive-sense single-stranded ribonucleic acid (RNA) type, and their ability to mutate rapidly makes the prescription of a standard drug unfeasible. There’s a chance that this disease won’t affect everyone, and because of the virus’s unpredictable nature, it may be devastating for those with weakened immune systems. As a result of its rapid spread, an early and precise diagnosis is considered a medical emergency. Reverse Transcription Polymerase Chain Reaction (RT-PCR) and radiography images (x-rays and CT scans) are being employed to detect COVID-19 [2]. RT-PCR determines whether viral RNA is present in a patient’s sample. The main disadvantage of the RT-PCR method is that it only locates and identifies the presence of viral RNA, which means it might misclassify a patient who has recovered from the llness [3]. The RT-PCR test takes 3 to 6 hours to complete and must be performed numerous times to obtain an accurate diagnosis. Currently, most of the methods of healthcare institutions to identify COVID-19 patients are not fast enough to prevent the disease from spreading to more people. The Delta variants of concern are the subject of significant worldwide interest right now, as they are causing a large number of COVID-19 cases around the world and are linked to vaccine failures [4]. There are notable differences between patients infected with variants Alpha, Lambda, Mu, and Delta. As a result, there is a need to utilize a computer-assisted technique that can automatically recognize various forms of variants. With the present epidemic, which is progressively affecting the general population, time and effectiveness of service are critical, therefore, most health organizations employ cloud technologies to store, analyze, and visualize all patient records. Artificial Intelligence (AI) is the development of computer systems with intelligence similar to humans, such as learning from knowledge, recognizing patterns, and making autonomous decisions [5]. Convolutional Neural Networks have recently emerged as the most important driver of biomedical research [6, 7]. Deep learning algorithms have been extensively applied in medical image analysis applications such as skin cancer classification [8], breast cancer detection [9], EEG-based diagnosis [10], and brain illnesses [11, 12]. Because COVID-19 includes the screening of chest X-rays, deep learning-based diagnosis of the lungs can help radiologists detect symptoms in a potential patient quickly and precisely. Researchers have been hard at work developing effective Computer-Aided Diagnosis (CAD) tools for diagnosing the COVID-19 virus from medical images such as X-rays and CT scans [13–15].

The main objective of this paper is to present a new proposed unsupervised multiple-image database fusion learning algorithm to diagnose lung infections on chest X-ray images. There are many challenges we face, such as irrelevant and redundant images in deep learning models, so we aim to create a benchmark dataset of COVID-19 chest radiograph images to test the classification performance of various CNN models. This article also aims to explore the use of transfer learning using the Inception V3 model and analyze the available datasets and their distribution. Also to perform data cleaning and normalization to improve the performance of the deep learning model utilized in their fusion. Additionally, the paper aims to use t-SNE for dimensionality reduction and visualization of high-dimensional data with tunable perplexity to produce an optimized version of the fusion. In general, the objective of the article is to provide a comprehensive framework for diagnosing lung infections using chest radiographs and to improve the accuracy, efficiency, and reliability of the deep learning model. The main contribution of this paper:

- Propose a new unsupervised multiple-image database fusion learning algorithm for diagnosing lung infections in chest X-ray images.
- The algorithm utilizes cloud-based advanced data to obtain an initial set of COVID-19 imagery databases and uses the MD5 image hash as a duplication removal criterion.
- The paper also discusses the Inception V3 model for transfer learning and explores data characteristics and visualization techniques using the t-SNE algorithm.
- The proposed algorithm aims to address the issue of redundant and irrelevant images in machine learning models.
- The suggested final version of the balanced dataset has been verified for a multi-class recognition issue, with a diagnostic accuracy of 98.48%.
- The final dataset of COVID-19 chest X-ray images can be used as a benchmark dataset to test the classification performance of various CNN models.

The rest of the paper is organized as follows: Related works section gives an overview of relevant research on COVID-19 detection in X-ray images. The selected datasets and study techniques are discussed in depth in Multiple image database fusion and production section. The Final Version of dataset setup and data generation is discussed in Exploratory data analysis section. The experimental setup and results are presented in Experiments findings section. Work conclusion and future directions section concludes with suggestions for future work.

## Related works

Deep learning approaches were successfully applied to X-ray images for COVID-19 diagnosis, yielding intriguing findings in terms of accuracy, sensitivity, specificity, and the Area Under the Receiver Operating Characteristic Curve (AUC). In [16], For addressing the pandemic, the authors proposed a software detection technique based on chest X-ray images. The model was created using many pre-trained networks and their combinations. The approach detects COVID-19 using characteristics collected from pre-trained networks, a sparse autoencoder for dimensionality reduction, and a Feed-Forward Neural Network for output production. The model was trained using 504 COVID-19 scans and 542 non-COVID-19 scans from two publically available chest X-ray imaging datasets. Using the combination of InceptionResnetV2 and Xception, the approach was able to attain an accuracy of 0.95% and an AUC of 0.98%. Analyses of results have shown that using a sparse autoencoder as a dimensionality reduction strategy enhances the model’s overall accuracy. A simultaneous deep learning CAD system based on the YOLO predictor was presented in [17], which can identify and diagnose COVID-19 while distinguishing it from eight other respiratory disorders. Using two independent datasets of chest X-ray images and COVID-19, the CAD system was evaluated using five-fold tests for the multi-class prediction issue. An annotated training set of 50,490 chest X-ray images was used to train the CAD system. The suggested CAD predictor was used to identify and classify areas on whole X-ray images with lesions presumed to be attributable to COVID-19, reaching overall detection and classification accuracies of 96.31 % and 97.40 %, respectively. Most test images from COVID-19 and other respiratory disorder patients were properly predicted, with an average Intersection over Union (IoU) of more than 90%. Deep learning regularizers of data balance and augmentation improved COVID-19 diagnostic performance by 6.64 % and 12.17 %, respectively, in terms of overall accuracy and F1-score. Authors in  [18] presented three distinct Big Transfer (BiT) models for the diagnosis of patients afflicted with coronavirus pneumonia using X-ray radiographs in the chest: DenseNet, Inception V3, and Inception-ResNet V4. They performed models using 5-fold cross-validation, which revealed that the pre-trained DenseNet model had the best classification effectiveness of the two models provided, at 92 % (83.47 % accuracy for Inception V3 and 85.57 % accuracy for the Inception-ResNetV4 model). In [19] the authors compared the chest x-ray scans of patients with COVID-19 with those of healthy participants. They examined the performance of deep learning-based CNN models after cleaning up the images and using data augmentation. They compared the accuracy of the Inception V3, Xception, and ResNeXt models. 6432 chest x-ray scan samples were acquired from the Kaggle repository to assess the model performance, 5467 were used for training, and 965 for validation. When identifying chest X-ray images, the Xception model has the highest accuracy of 97. 97 % compared to other models.

## Multiple image database fusion and production

In order to perform a valid data aggregation using multiple imagery databases collected from various sources using different scanning devices, the hash technique (details in Hashed distributed stochastic neighbour embedding (HDSNE) section) is used first to perform the first phase via removing duplicated and empty images. This produces the first clean version of our fused database, then t-SNE (see Hashed distributed stochastic neighbour embedding (HDSNE) section) can be applied to reach the compact, described as a perfectly balanced version of the fused database, which has an equal number of instances per class constrained to the number of instances in the smallest class.

### Available data and materials

**Fig. 1:**
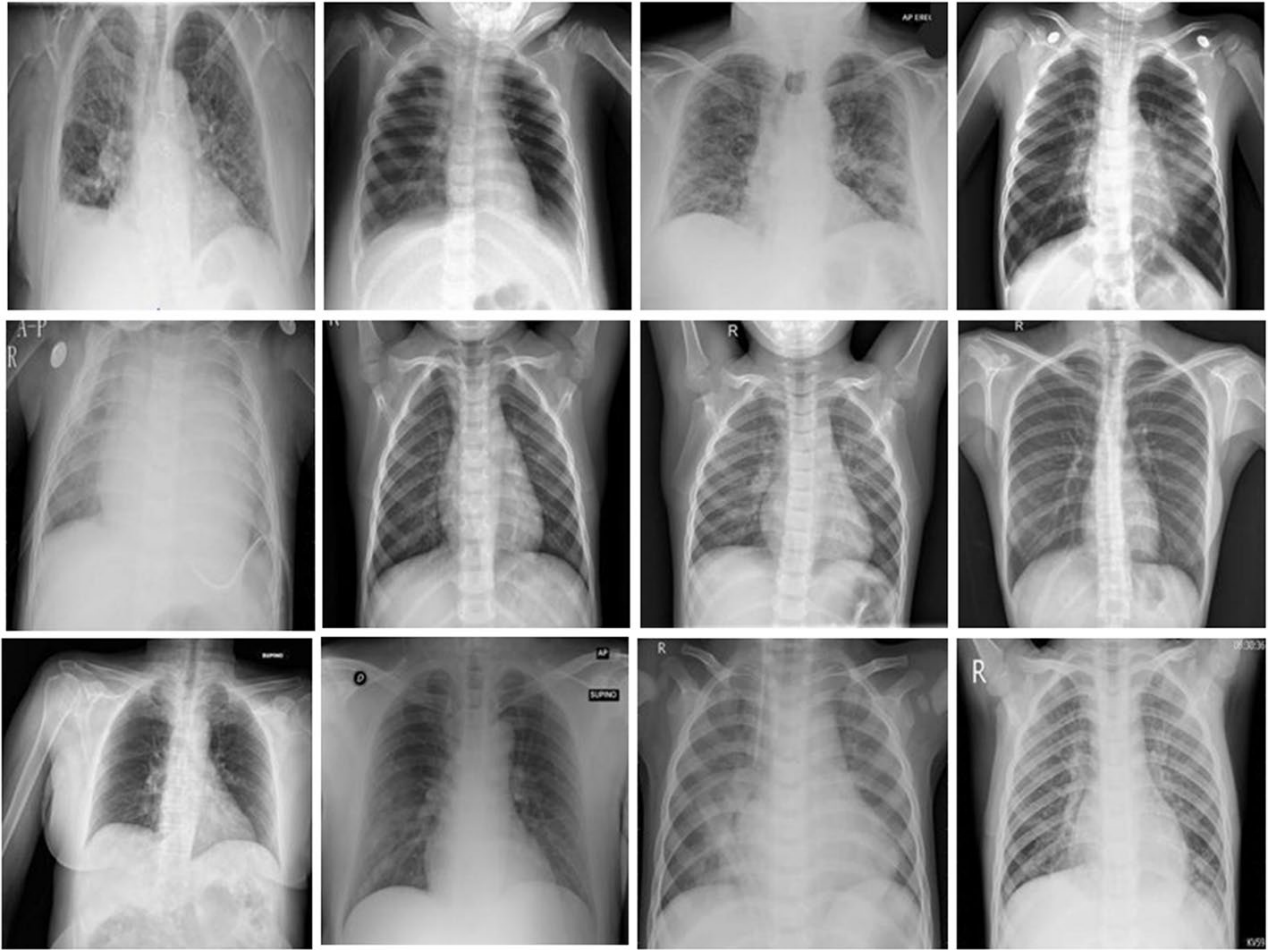
Random Samples of X-ray images of frontal chest cases from the DB^1^ to DB^6^ databases

This study presents a system for classifying frontal chest X-ray images into COVID-19, phenomena, and, no lung pathology (normal) for the purposes of the experiments. We combined the use of several available datasets with the addition of a new one comprising negative COVID-19 cases. In this research, X-rays were obtained from the sources shown in Table 1 with different resolutions. For each source, this table shows the distribution of the frontal view of chest radiography X-ray images across three classes: normal, patients infected with COVID-19 positive cases, and patients infected with various types of bacterial and viral pneumonia, such as MERS, ARDS, and SARS. In this paper, the proposed dataset is created by combining the following six publicly available frontal chest X-ray images (random samples from all datasets as shown in Fig. 1):

1. Imagery Dataset is split into three categories, each of which comprises different image formats [20]. It is available on the Kaggle website^1^; it has a total of 313 images containing viral pneumonia and normal chest X-rays divided into test and training folders of various dimensions. The University of Montreal has granted permission to use the images and data collected.
2. Radiography Database [21] of positive chest X-ray images, COVID-19, as well as viral pneumonia and normal images. This data was collected from the COVID-19 Dataset of the Italian Society of Medical and Interventional Radiology (SIRM) [26], the Novel Corona Virus 2019 Dataset created by Cohen on GitHub [27] and images from 43 different publications. A Radiography dataset has a file (PNG) format and has a dimension of $1024\times 1024$ pixels.
3. Patient dataset in [22] of chest X-ray images for COVID-19 positive cases from the available Kaggle website 2, along with normal and COVID-19 images, is used in our study. The dataset has two classes of COVID-19 positives, and no finding cases in this version. This dataset has different file formats and different resolution dimensions.
4. COVID-19 X-ray dataset [23] of chest X-ray images for pneumonia patients, as well as normal cases data images, are on the Kaggle website 3. The dataset has two classes with different resolution sizes and file formats: JPEG and PNG.
5. Patients lungs dataset [24] of chest X-ray images for COVID-19 cases as well as normal cases data images from the Kaggle website 4. In this current edition, the dataset has two classes with different resolution sizes and file formats: JPEG and PNG.
6. COVID-19 CoronaHack dataset [25] has two classes of X-ray radiographs in the CoronaHack dataset: normal and pneumonia patients with various causes. The dataset contains an imbalanced data collection and various resolution sizes in JPEG and PNG file formats. The Italian Society of Medical and Interventional Radiology (SIRM) prepared this image collection [26]. The authors gathered the radiological images from a variety of trustworthy sources, which are available online in [25]. The data comprises a collection of normal and infected patients for many categories, such as viral infection (cases with COVID-19), Severe Acute Respiratory Syndrome (SARS), bacterial infection (Streptococcus), and Acute Respiratory Distress Syndrome (ARDS).

**Table 1:** The collected COVID-19 X-ray images databases for final data aggregation (“-” meaning that this class does not exist)

Num	Data Name	Data Source	Normal	Pneumonia	COVID-19	Resolution	Total
1	Imagery	DB^1^ [20]	90	90	137	Varied	317
2	Radiography	DB^2^ [21]	1341	1345	209	$1024\times 1024$	2905
3	Patient	DB^3^ [22]	140	-	144	Varied	284
4	X-ray	DB^4^ [23]	94	94	-	Varied	188
5	Patients Lungs	DB^5^ [24]	28	-	70	Varied	98
6	CoronaHack	DB^6^ [25]	1574	4276	58	Varied	5908
Total	6	6	3267	5805	618	Varied	9690

## Hashed distributed stochastic neighbour embedding (HDSNE)

Due to the high number of irrelevant and redundant images, optimal imagery data use through machine learning is a big issue [28]. Any machine learning model spends a large amount of time, complexity, and expense getting complete training images from all the raw collected data, most of which are duplicates. Despite that, the duplicate data can affect the performance of the model if it uses similar features during training and doesn’t focus on essential features that differ from the model. To address this, the effective hashing algorithm MD5 [29, 30] is the ideal approach for removing image duplication. It generates a unique hash value for each image in the database, ensuring that we can properly delete images with the same hash value. The Algorithm 1 contains the pseudo-code for the algorithm utilized to provide our proposal. The querying method for a padding vector image M with multiples of i-bit width is as shown in Eq. 1:

1 $$\begin{aligned} H_{i+1} = f (H_{i}, M_{i}), 0 \leqslant i \leqslant t - 1. \end{aligned}$$

**Figure:**
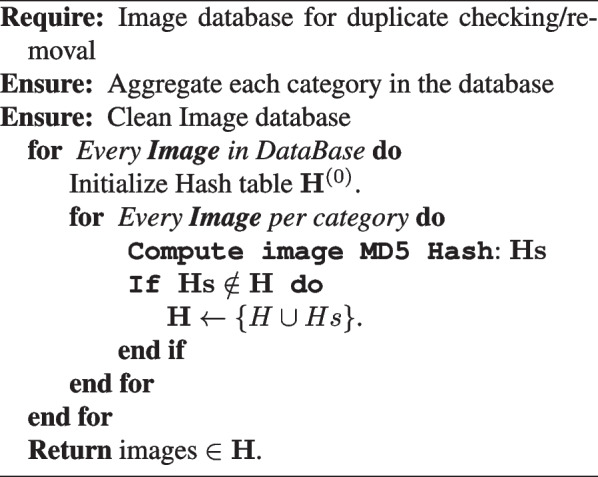
**Algorithm 1** HDSNE Stage 1: The proposed image duplicate detector

Where $H_{0} = IV_{0}$ is the hash function’s initial value of the first image of data. The next equations from Eqs. 4 to 7 represent the key mathematical concepts of the t-SNE algorithm applied to aggregated data. It is a useful algorithm for representing high-dimensional data into a 2D or 3D point map where each high-dimensional data sample (image in our case) is located, which is a key for selecting a specific subset of images according to their projection distance from an estimated class center point. Given an initial image dataset $\mathcal {I}_{Num}$ composed of *NUM* images where $\mathcal {I}_{Num}=\left\{ img_1, img_2,\ldots ,img_{NUM} \right\}$. Equation 3 represents the conditional similarity between two images, where $P_{ij}$ represents the similarity between image *i* and image *j*, and $\Delta _{ij}$ (Eq. 2) represents the difference between the feature vectors of the two images. The similarity is calculated using a Gaussian distribution with a variance of σ^2^.

2 $$\begin{aligned} \Delta _{ ij}=img_i-img_j ,\quad i \ne j \end{aligned}$$

3 $$\begin{aligned} \mathbf {\Delta }_{ij}=1+\left\| \Delta _{ij} \right\| ^{2}. \end{aligned}$$

It recursively requires calculating $\Delta$ for two images plus the square determinant of $1+\Delta$, then iteratively starts with computing the image pair-wise affinity probability as in Eq. 4. It defines the similarity between two images in the opposite direction, where $Q_{ij}$ represents the similarity between image *j* and image *i*. It is calculated using the inverse of the difference between the feature vectors of the two images.

4 $$\begin{aligned} \mathbb {P}_{ij} = p_{img_j\vert img_i}=\frac{\exp (-\Vert \Delta _{ij}\Vert ^2/2\sigma ^2)}{\sum _{k\ne i}\exp (-\Vert \Delta _{ik}\Vert ^2/2\sigma ^2)}. \end{aligned}$$

This measures how close a Gaussian distribution is centered on a certain variance $\sigma ^2$. This variance varies for each individual image, with images in dense areas receiving a lesser variance than those in sparse areas. In Eq. 5, the distance between two similarity maps is calculated using the inverse of the difference between the feature vectors of the two images. t-SNE derives its cost function ($\mathbf {C_F}$) from the Kullback-Leibler (KL) divergence resulting from the paired affinities in the space (${P}_{ij}$) and the similarities in the embedding (${Q}_{ij}$). During the optimization procedure, the ($\mathbf {C_F}$) is reduced.

5 $${\mathbb{Q}}_{ij}=q_{img_i\vert img_j}=\frac{({\overrightarrow\triangle}_{ij})^{-1}}{\sum_{k\neq j}({\overrightarrow\triangle}_{ik})^{-1}}.$$

The gradient of the cost function with respect to image *i* is calculated by Eq. 6, where $\frac{\delta C}{\delta img_i}$ defines the gradient, $P_{ij}$ and $Q_{ij}$ represents the similarity between image *i* and image *j*, and $\Delta _{ij}$ is the difference between the feature vectors of the two images.

6 $$\begin{aligned} \frac{\delta C}{\delta img_i}=4\sum _j(\mathbb {P}_{ij}-\mathbb {Q}_{ij})\Delta _{ij}. \end{aligned}$$

Finally Eq. 7 calculates the similarity between two images using the t-SNE algorithm, where a t-distribution with a degree of freedom of 1.

7 $$\mathbb{Q}(\Delta_{ij})=\Delta_{ij}(1+({\overrightarrow\triangle}_{ij}))^{-1}.$$

The pseudo-code of the t-SNE algorithm is explained in Algorithm 2.

**Figure:**
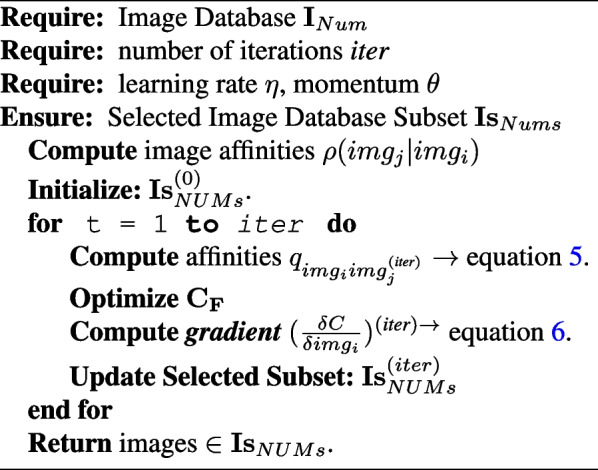
**Algorithm 2** HDSNE Stage 2: Flexible aggregation with a crispy constraint on the output of Algorithm 1

**Figure:**
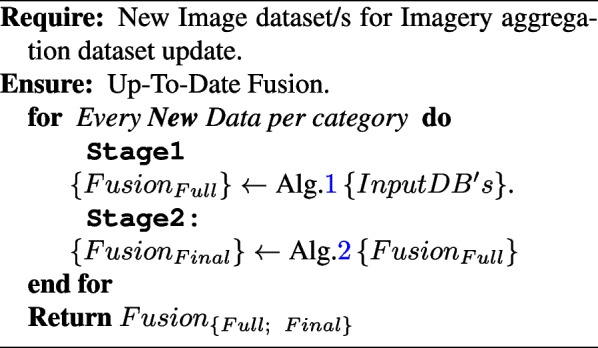
**Algorithm 3** HDSNE Final Production of Unique Image Database from Multiple Databases

**Fig. 2:**
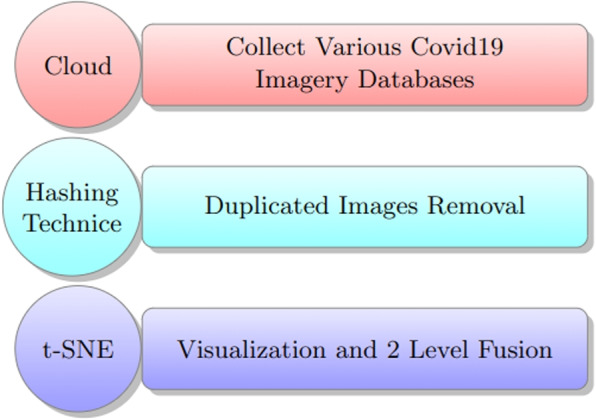
Collecting data aggregation and analytic methodology

The study describes a new unsupervised multiple-image database fusion learning algorithm for diagnosing lung infections in chest X-ray images. The algorithm utilizes cloud-based advanced data to obtain an initial set of COVID-19 imagery databases and uses the MD5 image hash as a duplication removal criterion. The recent availability of cloud-based advanced data has transformed the cyber into a data mine. The cloud is the source from which we obtained our initial set of COVID-19 imagery databases. Due to the necessity of data inter-integrity for mobile model production, which hopefully will perform well in reality, an MD5 image hash is used as image duplication removal criteria (see Hashed distributed stochastic neighbour embedding (HDSNE) section and Algorithm 1) bypassing only images with a unique hash value from the initial image population obtained from the cloud. Algorithm 2 presents a flexible collection with a crispy constraint, which is applied recursively to produce a perfectly balanced image database with the number of images per class equal to the number of images in the minor class and to get the final production of a unique image database from many databases (see Algorithm 3).According to Algorithm 3, the update of ${NUMs}^{(iter)}$ is done by computing the gradient of the $\mathbf {C_F}$ with respect to the image *i*, and then updating the selected subset $Is_{(iter)}$ using Eq. 6. The algorithm iterates over the number of subsets *NUMs* and returns the images in $Is_{(NUMs)}$. The proposed data framework is represented by the other meaning of a graphical pipeline, as in Fig. 2.

### Inception V3 deep learner

By their nature, deep learning models need a lot of data. Furthermore, since the COVID-19 data set is relatively small compared to normal deep learning datasets, the notion of transferring learning can be employed to help decision-making. Transfer learning is based on the idea of transferring information from one domain to another using previously taught weights. During other domain training, weighing arrays of many layers are traditionally frozen from the start, and only the remaining layers are modified. When both diseases have an overlap area in the case of different lung infections with low-level characteristics such as their structure, number, placement, and distribution, the transfer learning model is able to classify them effectively [31]. The trained weights from the ImageNet dataset were utilized to establish our model weights, but none of them were frozen since the ImageNet and COVID-19 datasets correspond to nonoverlapping domains. As a consequence, all classes are still started with weights that are more essential than random initialization and are sensitive to learning throughout the training phase. We focused on the Inception V3 model, which is commonly used for transfer learning and is publicly accessible in packaged form via trusted public libraries such as Keras, to find the best-suited model for our research. These models are conveniently included in the Keras API, and each one enables transfer learning [32] through pre-implementation functionality for ImageNet weights [33]. Inception V3 [34] is a pre-trained model architecture designed to maximize the use of computational resources inside the network by expanding the network’s depth and breadth while maintaining the same computation procedures. The term “Inception modules” was invented by the network’s designers to represent an efficient network structure with skipped connections that can be used as a construction component. To decrease dimensionality and complexity, each Inception module is replicated spatially by stacking with occasional max-pooling layers. The Inception V3 model is used to extract features. It is Google’s pre-trained model, which has been trained on over 1.4 million images and over 1,000 classes. The Inception V3 model is widely used in image detection models that use convolutional neural networks to extract image features.

## Exploratory data analysis

**Fig. 3:**
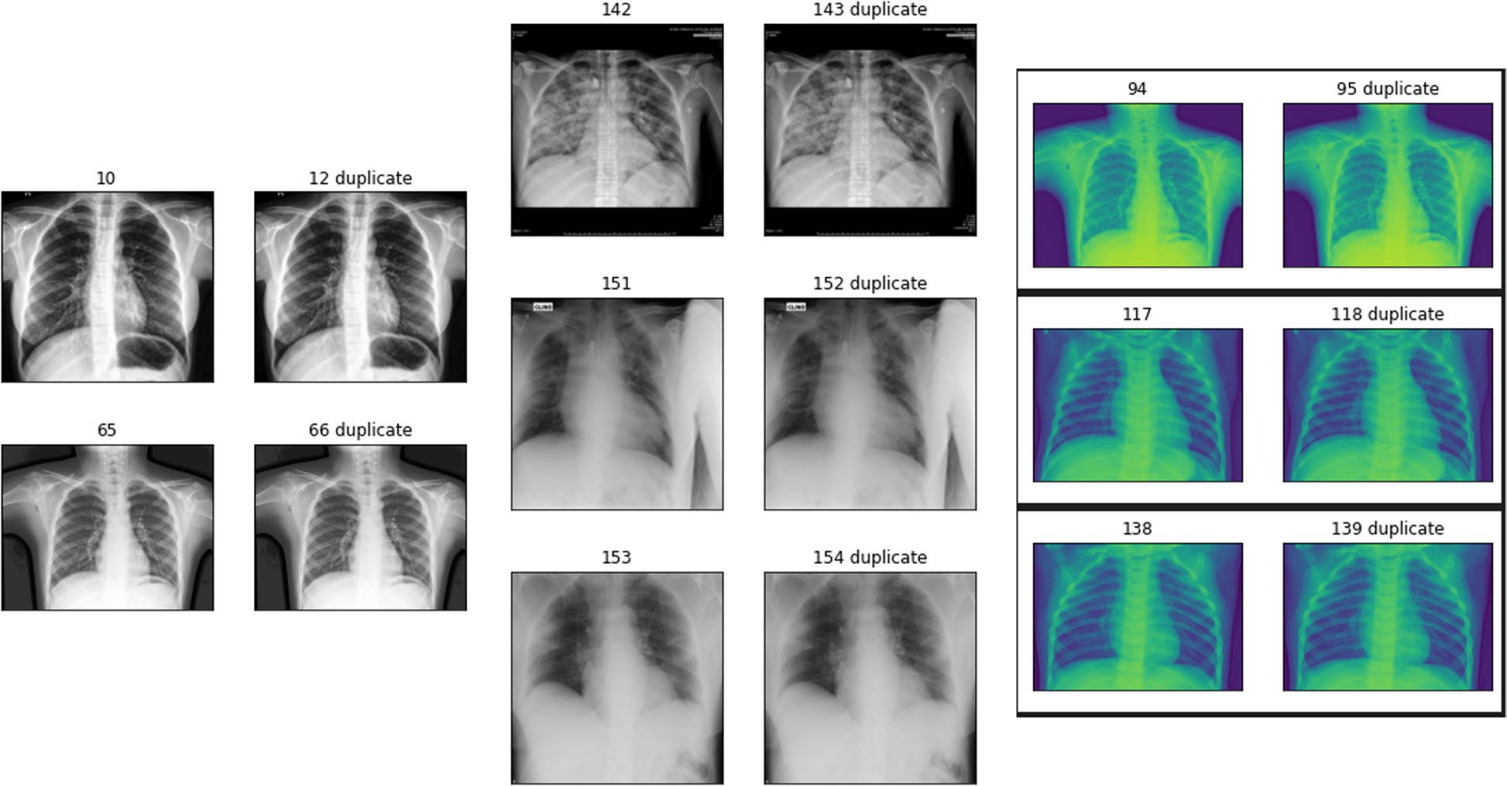
A sample of the detection process of duplicates using the MD5 hash algorithm

In the deep learning process, pre-processing is a crucial stage. Data collection techniques are frequently approximated, with out-of-range estimation, difficult information mixtures, and missing characteristics. Exploratory information processing is set up for primary preparation or further examination. Data preprocessing is the process of preparing raw data so that it can be used by an AI model. It is the first and most important step in making an AI model more robust. Data cleaning and normalization techniques are used to remove abnormalities and normalize the data. It requires the creation of a structure that can be easily utilized to create a model. Duplicate images in the dataset pose challenges for two purposes: they introduce a bias in the dataset, giving the deep neural network more opportunities to learn specific patterns of duplicate copies. Although data points in the dataset are frequently believed to be independent and equally distributed, this affects the model’s ability to generalize to new images outside of what it was trained on. Researchers commonly aim to eliminate these data duplicates before training a convolutional neural network. Second, manually detecting duplicate images in a dataset requires time, is error-prone, and doesn’t perform well with large image datasets. As a result, we require a way to detect and eliminate duplicate images from our data automatically. For that, we will detect and remove duplicate images in a proposed COVID-19 dataset presented in Table 1. The image hashing algorithm is the proposed image duplicate detector, as presented in Hashed distributed stochastic neighbour embedding (HDSNE) section as follows: First, the model performs duplicate image detection in the six datasets, detecting 634 duplicate images from DB^6^, 21 duplicates from DB^2^, and 4 duplicates from DB^1^ and DB^4^ as shown in Fig. 3.

**Fig. 4:**
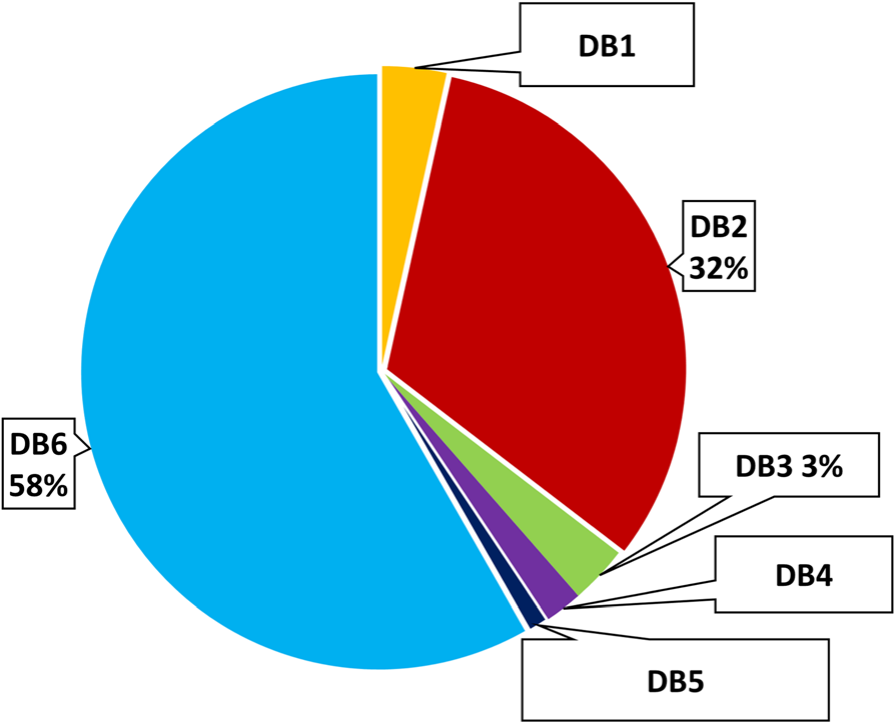
Distribution data for all merged X-ray images

Data analysis includes Data cleaning, transforming, and modeling to identify useful information for effective decisions is defined as data analysis. It considered a variety of data distributions between the data classes for all data in Fig. 4. The model’s effectiveness is impacted by the amount of variation in the three classes. The duplicate detector is then run a second time to remove the real duplicates from the given dataset. Table 2 presents all the cleaned data from the COVID-19 X-ray image after deleting all duplicates using an MD5 hash algorithm. Then, cleaning up unnecessary data by eliminating 114 out-of-scope CT images that degrade model performance. Finally, we’ll review the outcomes of our work in Table 3.

**Table 2:** A Cleaning data of COVID-19 X-ray image after deleting duplicates using an MD5 hash algorithm (“-” meaning this category does not exist)

Num	Dataset Name	Normal	Pneumonia	COVID-19	Total
1	DB^1^ [20]	88	89	136	313
2	DB^2^ [21]	1340	1340	204	2884
3	DB^3^ [22]	140	-	144	284
4	DB^4^ [23]	94	94	-	188
5	DB^5^ [24]	28	-	70	94
6	DB^6^ [25]	1356	3859	45	5260
	Total	3046	5382	599	9027

**Table 3:** Integrate clean data after removing out-of-scope anomalies and duplicates of images and presents the proposed final dataset

Dataset	Normal	Pneumonia	COVID-19	Sum
All data	3046	5382	599	9027
Duplicates	221	423	19	663
All DBs	2825	4959	580	8364
Final dataset	441	441	441	1323

### Statistical data characteristics exploration

**Fig. 5:**
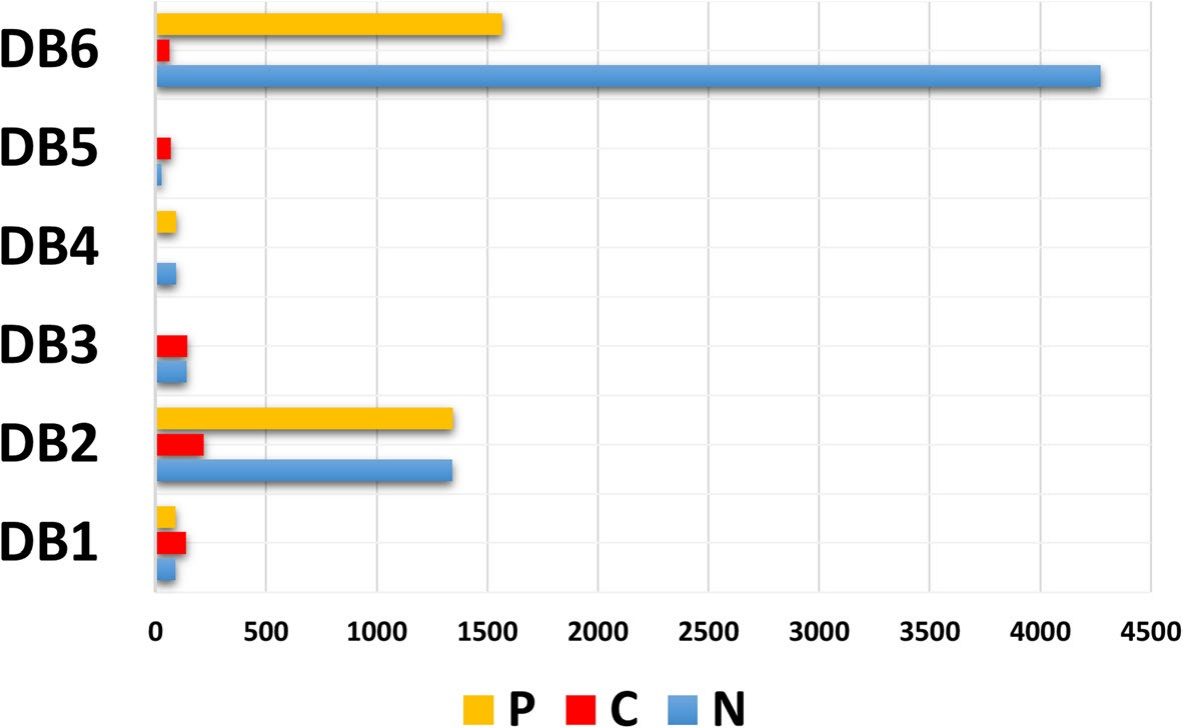
The data analysis of COVID-19 X-ray datasets (P = Pneumonia, C = COVID-19, N = Normal)

**Fig. 6:**
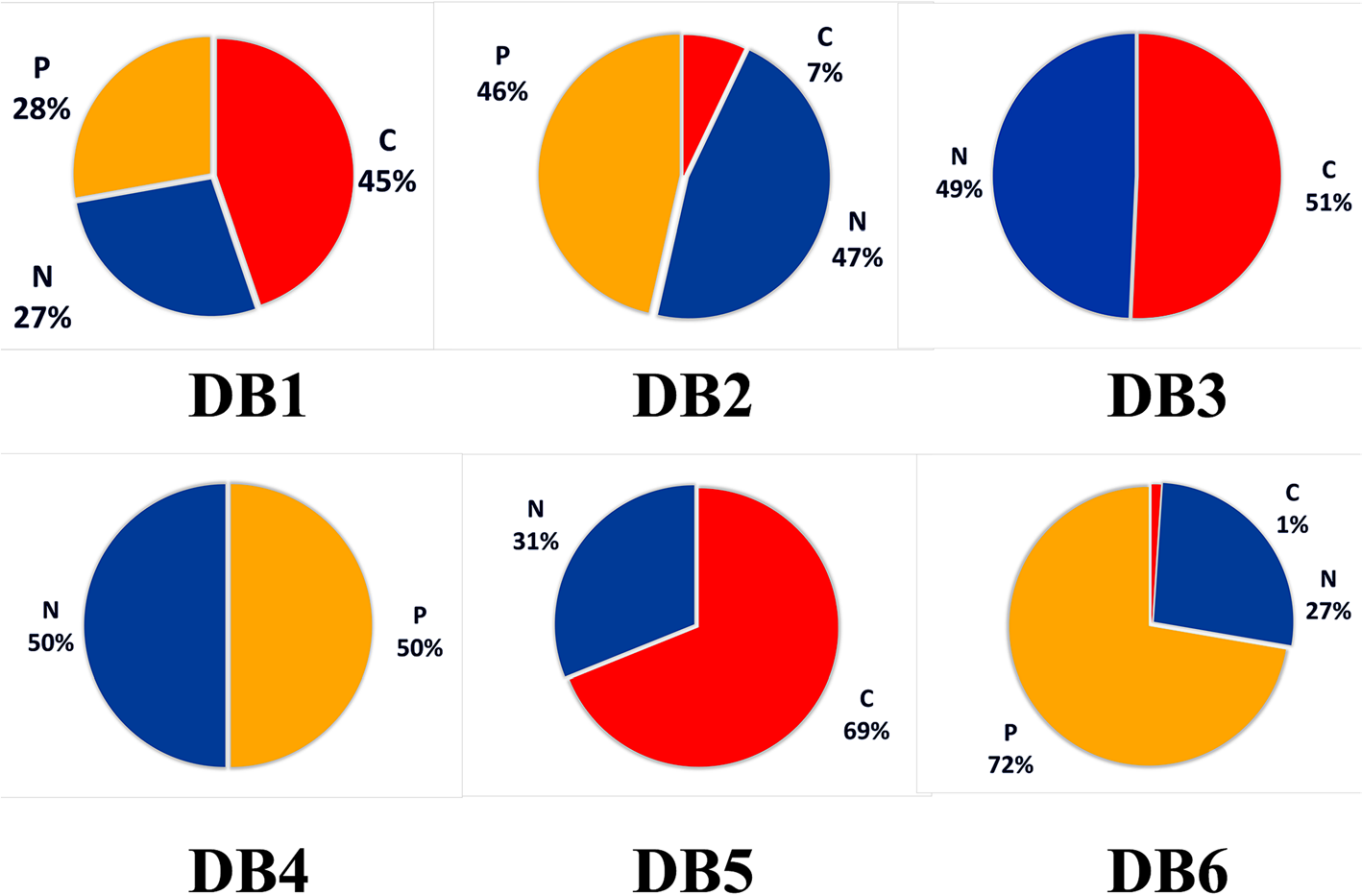
COVID-19 DB^1^ to DB^6^ classes distribution

Data is the fuel for modern computing. Whether it is the medical field or the retail market, data is the most precious thing in every field. Recent AI techniques are mostly followed by data-driven approaches. Deep learning-based algorithms almost fully depend on the dataset. As shown in Table 1 and in Fig. 5 there is a variety of DB$^1$ data distribution between the dataset classes in the training and testing set. However, it can be observed that the COVID-19 class has about 45% of the data. The variety of DB$^2$ data distribution between the dataset classes is quite large. However, it can be observed that the COVID-19 class has about 7% of data, and the COVID-19 class in DB$^6$ has about 1% of data, as shown in Fig. 6. As presented in Figs. 5 and 6, a convergence of DB$^3$, DB$^4$, and DB$^5$ data distribution ratios between dataset classes is founded. The amount of variance in the three data classes represents a major challenge in model performance. Table 1 and Fig. 5 demonstrate that the DB$^1$ data distribution differs across dataset classes in both the training and testing sets. Notably, the COVID-19 class contains approximately 45% of the data. The variety of DB$^2$ data distribution between the dataset classes is quite large. However, we can observe that the COVID-19 class has about 7% of data, and the COVID-19 class in DB$^6$ has about 1% of data, as shown in Fig. 6. In both Figs. 5 and 6, we can observe the convergence of data distribution ratios for DB$^3$, DB$^4$, and DB$^5$ across different dataset classes. Model performance is significantly challenged by the variance in the three data classes.

### Data representation and visualization

**Fig. 7:**
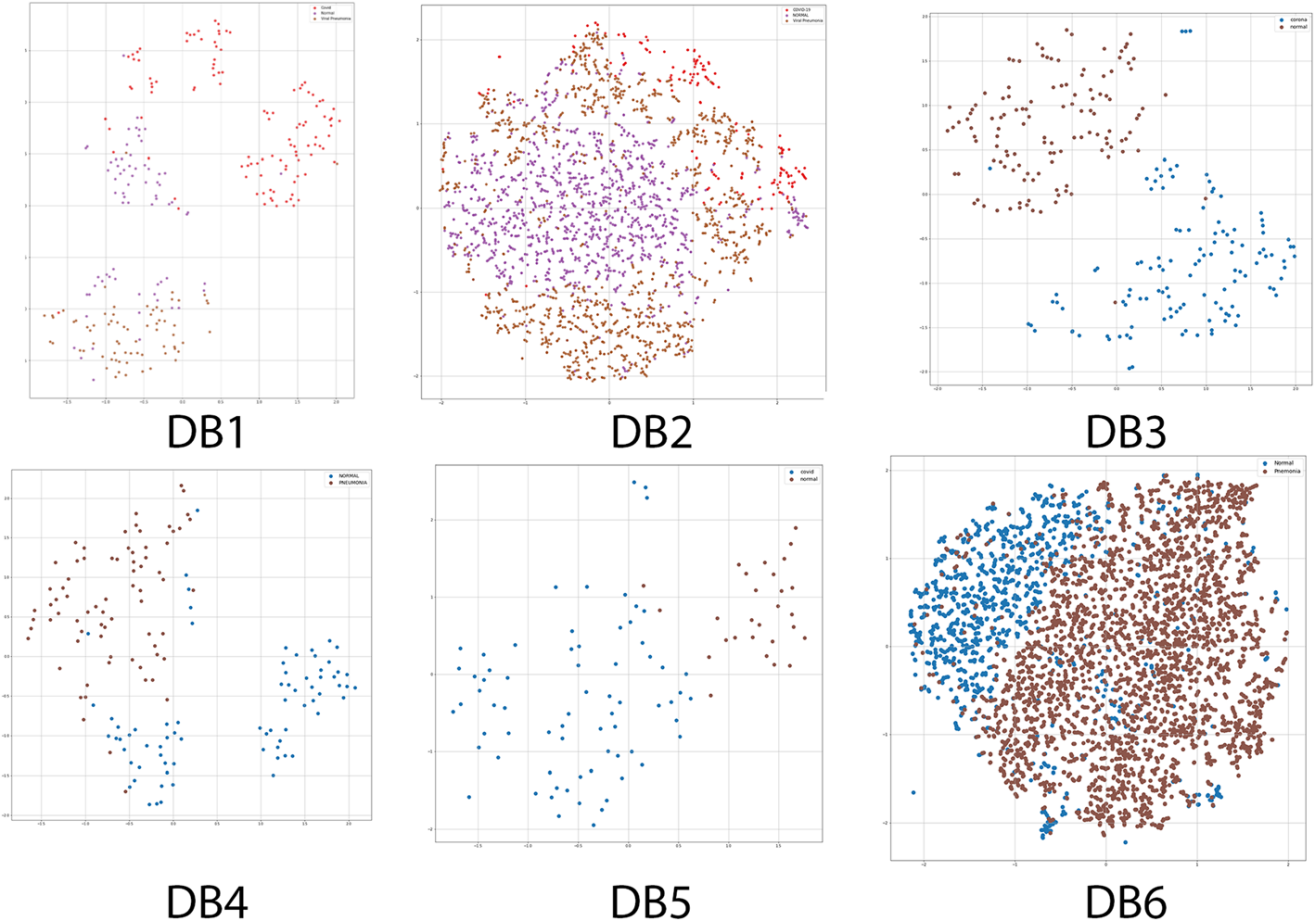
COVID-19 data visualisation using t-SNE with points from DB^1^ to DB^6^

Due to the database’s high dimensions, it could have minimized the high-dimensional feature space to a lower dimension, ignoring the highly linked characteristics. This phase is essential for class decomposition since it results in more homogenous classes, lower memory needs, and improved model efficiency. t-SNE is a dimensionality reduction algorithm that is highly suitable for visualizing high-dimensional datasets, such as those shown in Fig. 7. t-SNE reduces the divergence between two distributions: a pair-wise similarity distribution for the input objects and a pair-wise similarity distribution for the corresponding low-dimensional points in the embedding. Essentially, it looks at the proposed databases (Available data and materials section) that are fed into the algorithm and determines the optimal way to represent them with fewer dimensions by matching both distributions. The t-SNE dimension reduction approach was used, and the scikit-learn Python package was used to implement it [35]. The default scikit-learn hyperparameters (perplexity = 30, iterations = 1000, learning rate = 200) were used to tune the t-SNE hyperparameters. As a result of the proposed HDSNE algorithm in Algorithm 3, a new final version dataset is created for the final production of the data from the hash and t-SNE algorithms.

## Experiments findings

In this section, we first provide all the information about the experimental setup used and then evaluate six state-of-the-art COVID X-ray datasets, integrating all data and balancing the final dataset using the pre-trained Inception V3 model. The images were then normalized, scaled, and resized to $224\times 224$ pixels at 72 dpi [36, 37] to decrease the computational complexity. The prepared dataset is summarized in Table 3. The categorical cross-entropy loss we utilized is one of the most commonly used loss functions for deep neural network model training, especially in (multi-class) classification applications [38]. This loss function correlates to a probabilistic log-likelihood when applied to categorical data, resulting in advantageous estimation characteristics. During every trial, used 80% of the six datasets stated in Tables 2 and used the All DBs and the Final dataset presented in Table 3 for the training phase. Fully connected and Softmax layers are used for further detection. The data is then sampled for training and testing using a data generator. The remaining 20% of all experiments were then given to the prediction phase, and finally, the accuracy and loss were measured to evaluate the model training’s performance. A rectified linear unit is employed as the activation function (Relu). It is linear for all positive values and for all negative values to zero values. Because it is simple to calculate, the model takes less time to train. This function is employed because it avoids the vanishing gradient issue that other activation functions, such as sigmoid and tanh. It can be stated mathematically as described in the equation:

8 $$\begin{aligned} Q(\chi ) = \max (0, \chi ) \end{aligned}$$

Here, Q(.) is the function, 0 is the starting value, and $\chi$ is the input. The starting value is set to 0 since the Relu function returns 0 for all negative values. In the training phase, we used 30 epochs, a batch size of 16, and a learning rate of 0.0001 to make sure that all hyper-parameters were the same. The results of the COVID-19 X-ray image classification using the Inception V3 model, as presented in Table 4, provide valuable insights into the model’s performance across various datasets. The statistical analysis reveals important performance metrics such as accuracy, precision, recall, and F1-score, which help evaluate the effectiveness of the model in differentiating between classes within the datasets. Starting with the binary-class datasets, we observe varying degrees of performance. DB$^1$ achieved a high accuracy of 94% with balanced precision, recall, and F1-score values. Similarly, DB$^2$ showed an accuracy of 94% and balanced metrics. These results indicate that the model performed consistently well in accurately classifying positive and negative cases within these datasets. Moving on to DB$^3$, we observe a lower accuracy of 71% along with balanced precision, recall, and F1-score values. This suggests that the model achieved a moderate level of performance in accurately classifying the images in this dataset. Analyzing DB$^4$, we see an accuracy of 72.22% along with precision, recall, and F1-score values of around 72%. These results indicate that the inception V3 model achieved a relatively similar level of performance across these metrics. However, the overall performance in DB4 is slightly lower compared to the previous datasets. The balanced precision, recall, and F1 score suggest that the inception V3 model achieved consistent classification performance, but with a slightly higher misclassification rate and a high outlier of data. Moving to DB$^5$ predictions, we observe an accuracy of 89.47% along with relatively high precision, recall, and F1-score values. However, the F1-score is slightly lower compared to the accuracy, showing that the inception V3 model may struggle with classifying certain instances within this dataset. Overall, the model achieved good overall classification performance for DB$^5$, although there may be some imbalance in terms of precision and recall. For the multi-class datasets, DB$^6$ achieved an accuracy of 79.35% along with balanced precision, recall, and F1-score values of around 79%. These results indicate that the inception V3 model achieved moderate performance in accurately classifying the images in DB6. Balanced precision, recall, and F1-Score suggest relatively consistent performance in terms of positive and negative predictions. Considering the combined dataset (DB All), the accuracy drops to 69.3%. However, precision, recall, and F1 score show a relative value of around 70%. This indicates that the model encountered challenges in accurately classifying the images within the combined dataset, potentially due to the complexity of having multiple classes with varying characteristics. The low relative precision, recall, and F1-score suggest a relatively consistent performance in terms of both positive and negative predictions, with a lower accuracy rate. Finally, the proposed Final dataset achieved the highest accuracy of 98.48% along with high precision, recall, and F1-score values. These results indicate excellent overall classification performance for the Final dataset, demonstrating the model’s ability to classify COVID-19 X-ray images within this balanced dataset accurately. When it comes to classifying COVID-19 X-ray images from different datasets, it’s crucial to analyze the performance using statistics. The Inception V3 model can be used to assess the performance of the model across all datasets or evaluate specific datasets. While the binary-class datasets demonstrate higher accuracies and balanced metrics, the multi-class datasets pose additional challenges. However, the Final dataset benefits from a balanced distribution of samples and stands out with exceptional performance. The benefits of our balanced dataset, including mitigating class imbalance, improving feature learning, and enabling fair evaluation, contribute to the model’s success in accurately classifying COVID-19 X-ray images. The creation of the Final dataset using hash for deduplication and t-SNE for data representation offers significant benefits over the combined dataset. The elimination of duplicate entries through the hash function ensures data integrity and reduces biases that may arise from redundant information. The use of t-SNE enables better data visualization, aiding in the identification of clusters, outliers, and underlying patterns within the dataset. These benefits contribute to a more accurate and insightful representation of the Final dataset, enhancing subsequent modeling and classification tasks.

**Table 4:** The overall performance classification results of COVID-19 datasets using the Inception V3 model

Dataset	Classification Type	Accuracy	Recall	Precision	F1-score
DB$^1$	Multi-class	94	94	94	93
DB$^2$	Multi-class	94	94	94	94
DB$^3$	Binary-class	71	71	71	71
DB$^4$	Binary-class	72.22	72.5	72.22	72.14
DB$^5$	Binary-class	89.47	90.79	89.47	88.51
DB$^6$	Multi-class	79.35	78.7	79.35	78.6
All DBs	Multi-class	69.3	83.61	69.3	70.08
Final dataset	Multi-class	98.48	98.5	98.48	98.48

In this study, we effectively measured the significance of differences in model performance between the “Final dataset” and the union of six datasets using hypothesis testing. In this case, we used a paired t-test [39, 40], a well-established method to compare two related groups, to determine whether observed variations in effectiveness metrics are statistically significant. The “Final dataset” was our target dataset, while the union of six datasets provided paired observations for direct comparison. By calculating *t*-values and corresponding *p*-values for each measurement, we quantified the strength of evidence against the null hypothesis and determined whether the observed differences in model performance are statistically meaningful or due to chance fluctuations [41]. The hypothesis testing framework utilized in this study ensures the robustness and dependability of evaluating the performance of our proposed model, contributing to the validity of our conclusions. In our evaluation, we utilized a paired t-test to compare the effectiveness metrics of the union of six datasets with those of the Final dataset. The paired t-test is a robust statistical method for determining whether there is a significant difference between two related groups, making it a suitable option for our scenario. The “Final dataset” represents a specific dataset of interest, whereas the six union datasets serve as paired observations, allowing us to analyze the performance variations between the two related groups for each measurement. The formula for the *t*-value in the paired t-test is given as in Eq. 9:

9 $$\begin{aligned} t = \frac{\text {mean of paired differences}}{\frac{\text {standard deviation of paired differences}}{\sqrt{\text {sample size}}}} \end{aligned}$$

where the Standard Error (SE) is calculated as presented in Eq. 10 the standard deviation of the dataset divided by the square root of its sample size (n = 6, in our case) for each measurement:

10 $$\begin{aligned} SE = \frac{\text {Standard Deviation}}{\sqrt{n}} \end{aligned}$$

In hypothesis testing, the *p*-value is a crucial statistical measure used to evaluate the evidence against the null hypothesis, as calculated by Eq. 11. It quantifies the likelihood of observing the observed test statistic (*t*-value) or an even more extreme value under the null hypothesis.

11 $$\begin{aligned} p = 2 \times P(T > |t|) \end{aligned}$$

where *T* is the t-distributed random variable with the appropriate degrees of freedom, *t* is the observed *t*-value, and $P(T > |t|)$ is the cumulative probability of the t-distribution with degrees of freedom, which represents the probability of observing a *t*-value as extreme or more extreme than the observed |*t*| under the null hypothesis.

**Table 5:** Comparison of performance metrics between “final dataset” and “all data” using a paired t-test

Measurement	Mean (All Data)	Final Dataset	SE (All Data)	*t*-value	*p*-value
Accuracy	83.057	98.48	3.928	3.92	0.0202
Recall	82.997	98.5	3.957	3.91	0.0210
Precision	82.997	98.48	3.957	3.91	0.0210
F1-score	85.377	98.48	3.131	4.19	0.0138

This table presents a comparison of performance metrics between the “Final Dataset” and the “All Data” using a paired t-test. Mean values and Standard Errors (SE) are provided for both datasets. The *t*-value and *p*-value are calculated to determine the statistical significance of the differences. The results indicate that all differences are statistically significant at the chosen significance level ($\alpha$ = 0.05)

In Table 5, we have provided the computed values for the t-test results, which include the mean and SE for each measurement in all six data sets as well as the proposed Final dataset. *T*-values were calculated based on the paired t-test formula for related samples. The “Statistical Significance” column indicates whether the *t*-value for each measurement is statistically significant at the alpha = 0.05 level for all measurements. All *t*-values are greater than the critical *t*-value (approximately 2.571 for a two-tailed test), indicating statistical significance. Therefore, the results suggest that the Final dataset exhibits statistically significantly higher performance in terms of Accuracy, Recall, Precision, and F1-score compared to all six datasets. According to the results as presented in Table 5, the *p*-values we calculated for each measurement indicate the likelihood of achieving the observed differences in means between the two sets of data. A small *p*-value indicates strong evidence against the null hypothesis. We discovered that all performance metrics have statistically significant differences between “Final Dataset” and “All Data.” The results indicate that our “Final Dataset” outperformed “All Data” across these metrics.

Finally, in Table 6 we present a comprehensive comparison of the accuracy and various performance metrics achieved by our proposed model against those of existing techniques using the same dataset. It is important to note that the other models as listed in Table 6 were trained using different quantities of images from various data sources, which were then combined with any of the proposed data for three multi-class classifications. On the other hand, our proposed model was exclusively trained using the final dataset, comprising images from all the data sources. Remarkably, our proposed model exhibited outstanding performance across all evaluated metrics, showcasing its effectiveness and superiority in the classification task. The results further emphasize the significance of utilizing the complete and unified dataset, which allowed our model to capitalize on the diverse information available from all data sources, leading to remarkable predictive capabilities.

**Table 6:** Comparison of our results with other existing models using the same data or merged with others

Dataset	Reference	Accuracy (%)	Precision (%)	Recall (%)	F1-score
DB$^1$	[42]	N/A	N/A	N/A	88.8
	[43]	87.99	88.0	86.0	87.0
	Ours	**94.0**	**94.0**	94.0	93.0
DB$^2$	[44]	93.02	N/A	N/A	N/A
	Ours	94.0	94.0	94.0	94.0
Different sources	[45]	87.02	N/A	N/A	N/A
	[46]	94.2	N/A	N/A	N/A
	[47]	95.0	N/A	N/A	N/A
	[48]	96.0	96.1	96.9	96.5
	[49]	92.4	N/A	N/A	N/A
	[50]	93.48	N/A	N/A	N/A
	[51]	89.855	91.410	97.320	95.512
	Ours	98.48	98.5	98.48	98.48

## Work conclusion and future directions

It is critical to identify COVID-19 individuals early in order to prevent the illness from spreading to others. In this work, we used chest X-ray images from normal, COVID-19, and pneumonia patients to propose a deep transfer learning-based technique based on the Inception V3 net to predict COVID-19 patients automatically. We presented a novel technique HDSNE based on the MD5 Hashing Algorithm to clear data duplicates and the t-SNE unsupervised learning algorithm to better depict the data distribution due to the difficulties of capturing continuous changes in COVID-19 X-ray data variants. The suggested final version of the balanced dataset has been verified for a multi-class recognition issue, with a diagnostic accuracy of 98.48%. The statistical t-test has confirmed the results, with significant *t*-values and *p*-values. It’s essential to highlight that all *t*-values are unquestionably significant, and the *p*-values offer indisputable proof against the null hypothesis. Additionally, it’s worth noting that the Final dataset outperformed all other datasets in diagnosing various lung infections with the same factors, across all metric values.

Our results suggest that, because of the obviously improved performance, radiologists will be better able to make clinical decisions. This research reveals how deep transfer learning algorithms can be utilized to discover COVID-19 at an early stage in order to detect it. The final dataset of COVID-19 chest X-ray images can be used as a benchmark dataset to test the classification performance of the various CNN models in future research. Future studies might include combining additional datasets and other kinds of COVID-19 images, such as ultrasound and CT scan data, as well as developing updated pre-trained models and convolutional neural networks.

## Data Availability

Authors declare the availability of the created data [52] under public access license in https://data.mendeley.com/datasets/nttrfkg644, source code, and any materials that can be accessed upon request.

## Footnotes

1 https://www.kaggle.com/pranavraikokte/COVID-19-image-dataset.

2 https://www.kaggle.com/wahib04/COVID-19-patient-xray-image-dataset.

3 https://www.kaggle.com/khoongweihao/COVID-19-xray-dataset-train-test-sets.

4 https://www.kaggle.com/nabeelsajid917/COVID-19-x-ray-10000-images.
